# Transsphenoidal pituitary surgery by microscopic or endoscopic approach: the still unsolved question of superiority

**DOI:** 10.1590/2359-3997000000219

**Published:** 2016-10-01

**Authors:** Eduardo de Arnaldo S. Vellutini

**Affiliations:** 1 Centro de Base do Crânio de São Paulo São Paulo SP Brasil DFV Neuro São Paulo. Centro de Base do Crânio de São Paulo, São Paulo, SP Brasil

Surgery has been the main treatment of pituitary tumors since the beginning of last century. Pioneers like Cushing, Hirsch, and Schloffer were able to foresee the advantages of the transsphenoidal approaches but had to abandon them due to technical difficulties, specially those related to surgical field lighting. Even though this method was maintained by Norman Dott, in England, and Gerard Guiot, in France, it was only in the beginning of the sixties that Jules Hardy, in Canada, brought the surgical microscope to the operating room.

Since then, the microscopic transsphenoidal approach became the gold standard for the treatment of pituitary tumors. Meanwhile, in the middle of the nineties, endoscopic surgery had already proven its efficacy in the treatment of nasosinusal diseases and, thus, its use in sellar diseases became a matter of time. The wide-angle view associated with a light source near the surgical field brought about a large number of possibilities, not only for pituitary surgery but also in the whole field of skull base surgery.

In the last years, the indications for endoscopic pituitary surgery have gradually overcome those for microscopic surgery. View of the surgical field, the Achilles' heel of Cushing almost a hundred years ago, is the main advantage of this technique. Surely, surgeons must adapt to the changes in the operating room, to the use of a video monitor and to a two-dimensional surgical view with a different anatomic perspective. However, those difficulties should be faced after proper laboratory training, and it should be considered that learning may probably be faster for a young than for a senior surgeon.

Endoscopy has also brought the rhinologist to the field of pituitary surgery, which can be seen as a great advantage. Results were improved, developing multidisciplinary cooperation, bringing about many technical innovations in the operative room, improving nasal functional results in patients, as well.

There still are controversies among pituitary surgeons about the superiority of endoscopic techniques (1,2). In this issue of the *AE&M, *Bastos and cols. (3), by means of a meta-analysis of the literature, tried to figure out which may be the best indication for the patient, presenting pooled data on surgical success, outcomes, and complications of both techniques. They did not find a significant differences between endoscopic and microscopic TSS in relation to pituitary tumor resection rates and achievement of biochemical control of functioning pituitary adenomas, but pointed out that, due to the low level of evidence and small number of observations, the results of their meta-analysis should not be viewed as a final proof of inferiority or superiority of one approach in relation to the other. Nonetheless, postoperative complications seemed to be less frequent with the endoscopic procedure. As a matter of fact, such comparisons are difficult to evaluate due to different number of cases (larger in microscopic series), distinct eras of pituitary surgery (series from different decades), and heterogeneous tumor subgroups of pituitary adenomas.

Nevertheless, endoscopic pituitary surgery has clearly changed some paradigms. Tumors located in the cavernous sinus, which were previously referred for radiotherapy (1), and tumors with large suprasellar extension, which were operated by a standard craniotomy (4), can now be removed by endoscopic transnasal extended approaches with good results and acceptable complications.

Medical science is an ever-changing area. A multicenter, randomized, trial of microscopic and endoscopic transsphenoidal techniques may determine the real advantages and disadvantages of each of them. Meanwhile, the pituitary surgeon should choose the best for the patient according to his/her expertise.
